# The Effects of Population Density on the Incidence of Developmental Deformities in Chemosensory Organs of Tobacco Hornworm Larvae (Lepidoptera: Sphingidae)

**DOI:** 10.1093/jisesa/ieaa062

**Published:** 2020-07-17

**Authors:** Frank Hanson, Elizabeth Stanwyck, Alexander Bohorquez

**Affiliations:** 1 Department of Biological Sciences, University of Maryland, Baltimore County, Baltimore, MD; 2 Center for Interdisciplinary Research and Computing, University of Maryland, Baltimore County, Baltimore, MD

**Keywords:** larval crowding, chemosensilla, chemosensory deformation, life history traits, larval culture

## Abstract

Cultures of *Manduca sexta* Johanssen in our laboratory were found to have larvae with missing or deformed mouthparts or antennae. Hypothesizing that these developmental deformities were caused by crowded rearing conditions, we reared larvae in four different population densities and recorded the incidence (% of larvae affected) and types of chemoreceptor deformities. Results showed that the incidence of these deformities was directly proportional to larval population density. Deformities of the maxilla and palp were the most frequent, followed by those of the antenna, epipharynx and maxillary styloconica. Life history traits of larval mass, food consumption, and rate of development were inversely related to larval density for both normal and deformed larvae. We discuss possible causes and mechanisms of these deformities and of changes to life history traits.

Larvae of oligophagous insects such as the tobacco hornworm, *Manduca sexta* Johanssen, rely primarily on their gustatory chemoreceptors for food selection. The main gustatory organs border the chewing mouthparts (mandibles). Dorsal to these mandibles and covering the upper portion of the oral cavity is a bi-lobed flap, the epipharynx (Fig. 1A, arrow); in each lobe is an extremely small sensory organ that is in continuous contact with the oral contents during feeding. The mouthparts just ventral to the mandibles are the bilateral maxilla (Fig. 1A, asterisk), each of which contains two small (~50 µ) styloconica and a palp (Fig. 1A inset, S and P, respectively). The maxillae are highly mobile and can be seen moving into and out of the bubble of plant juice two or three times per second while the animal is feeding. Each of the above sensilla plays a distinct role in acceptance of host plants or rejection of non-hosts (de Boer 2006). Bilateral ablation of all of these sensilla essentially eliminates the animal’s ability to discriminate between host and non-host plants; for example, chemosensory-ablated larvae will eat *Canna generalis*, a plant so deterrent that normal larvae would prefer to starve rather than eat it (Waldbaur and Fraenkel 1961, Hanson and Dethier 1973, de Boer and Hanson 1987, de Boer 1991). Unilateral ablation also results in a loss of discrimination, but to a lesser degree (de Boer 1991, Flowers and Yamamoto 1992).

**Fig. 1.**
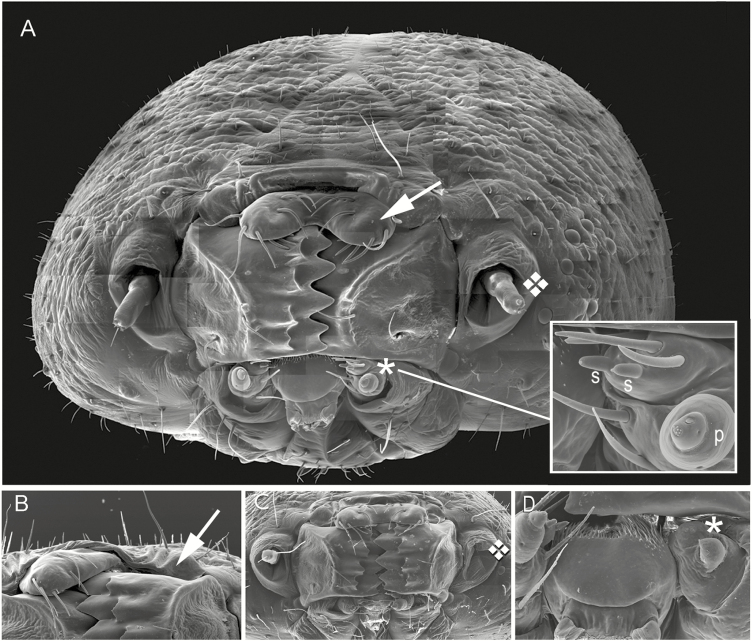
Normal and deformed or missing chemosensory organs. (A) Montage SEM of fifth-instar *M. sexta* larval head with normal chemoreceptors (although missing some ocelli). Arrow = epipharynx; diamond = antenna; asterisk = maxilla. Inset: enlargement of maxilla with styloconica (S) and palp (P). (B) Upper portion of larval head missing the left half of the epipharynx. (C) Middle portion of larval head missing the left antenna. (D) Lower portion of larval head showing deformed left maxilla (deformed palp and missing both styloconica).

The olfactory organs are the antennae (Fig. 1A, diamond) and possibly the maxillary palpi. The antennae have been shown to mediate behavioral attraction to humidity (Rowley and Hanson 2007) and food plants (Glendinning et al. 2009). The lateral maxillary styloconica have near-range (≤600 µm) olfactory capabilities as shown electrophysiologically (Städler and Hanson 1975), but it is not known whether this contributes to the animal’s overall olfactory capability.

Our research on the sensory basis of food selection has traditionally employed laboratory-reared larvae that were screened to exclude those with missing or deformed chemosensory organs described above. The incidence (% of larvae deformed) was noticeably variable, which prompted an investigation to determine the sources of this variability and what might be causing these deformities. After eliminating several potential sources such as genetic variation and hazards to eggs during shipment, we hypothesized that these deformities might be linked to population density in the larval cultures. Here, we present results of experiments to test this hypothesis and determine if any life history traits are also affected by population density. We discuss possible mechanisms and compare results with those from other species.

## Methods

### Insects

Eggs were obtained from the North Carolina State University Insectary’s *M. sexta* culture that was initiated in the 1960s and maintained on artificial diet (Yamamoto 1969). These eggs were shipped overnight in an insulated container, disinfected with 0.05% bleach solution, rinsed with distilled water, air dried, and placed on plastic mesh (window screen) on the floor of a 12-cm Petri dish. The cluster of eggs was surrounded by strips of tobacco hornworm diet (Yamamoto 1969; commercial source: Frontier Agricultural Sciences, Newark, DE) ~2 cm away to prevent inhibition of hatching. This culture was maintained for eggs and first instar in 16-h light and 8-h dark (L16:D8) and 24°C. Larval instars were determined by comparing head capsules with a ‘library’ of five sizes of head capsules accumulated over the years of rearing this insect. The transition to fifth (final) instar is conspicuous because the head capsule of the fourth-instar premolt appears as a bubble on the much larger fifth-instar head capsule developing behind it (‘bubblehead’).

### Population Density Experiments

Newly molted second-instar larvae were transferred to metal wire mesh platforms placed 2 cm above the floor of translucent polypropylene culture boxes (28L × 14W × 10H cm). Small holes in the sides provided passive ventilation. Each day, strips of diet in excess of anticipated consumption were placed on the platforms, and both the uneaten and replenishing food were weighed for the calculation of consumption. These weights were recorded daily for each box, as were larval counts (for the initial number, see below). Culture boxes were cleaned daily and sterilized with dilute chlorine bleach.

The first experiment consisted of 10 sequential trials, each of which was comprised of four simultaneous cultures of different population densities (10, 40, 60, or 100 animals per box) at L16:D8 and room temperature of 22°C, well within the range (20–30°C) for normal weight attainment (Moore et al. 2020). Upon reaching the final premolt condition at the end of the fourth instar, each larva was removed from the culture and transferred to a compartment (5.3 × 5.3 × 5 cm) of a larger transparent plastic box (32 × 21 × 5 cm) maintained at 30°C, 70% humidity, and L16:D8 without food or water until it molted into the fifth instar (usually that night). The larva was then weighed and examined under a dissecting microscope for deformed or missing chemosensory organs. Six classes of chemosensory deformities were recorded (Fig. 1): epipharynx (missing half); maxilla (missing, or deformed and without chemoreceptors); maxillary palp (missing or deformed); maxillary styloconica (one or both missing); antenna (missing or deformed); and multiple (more than one of the above). This information was recorded along with the date and its population density cohort; the animal was then euthanized or processed for scanning electron microscopy (SEM).

In the initial trial of this experiment, we only recorded whether each larva was normal or deformed; in the subsequent nine trials, we also recorded the status of each larva’s chemosensilla (normal or type of deformity) and the daily food consumption of each culture box. Thus, analyses involving total incidence of deformities used the data from all 10 trials (1,940 surviving larvae), whereas analyses concerning types of deformities or food consumption used data from only the last 9 trials (1,744 surviving larvae). (Two analyses, consumption and development, were limited to 1,743 and 1,734 larvae, respectively, because of missing data.) The effects of population density on both the incidence and type of chemosensory deformities, larval mass, development time, and food consumption were determined.

A second experiment was done to disentangle the effects of area population density, such as physical interactions between larvae on the feeding platform, versus the effects of volume population density, such as the headspace containing volatile chemicals such as pheromones. This experiment compared the incidence of deformities in cultures having the same populations and air volumes, but different areas of feeding platforms on which the larvae were reared. High-density cultures were reared on normal single feeding platforms (area = 392 cm^2^), whereas the low-density cultures spread the same number of larvae over three feeding platforms stacked 2 cm apart forming a three-level tier (tier platforms, area = 1,176 cm^2^). Thus, the latter had one-third of the area density of the former but, because all cultures used the standard culture box, they had the same volume density. A trial consisted of one high-density and three low-density cultures running concurrently at room temperature (~22°C) and light regimen (L16:D8). Three trials were conducted.

### Scanning Electron Microscopy

Fixation, ultrasonic cleaning in dilute detergent, and heavy metal deposition were performed on normal and deformed larval heads using standard techniques for SEM. Visualization was done on a JOEL model JSM 5600 scanning electron microscope.

### Analysis and Statistics

**Fig. 2.**
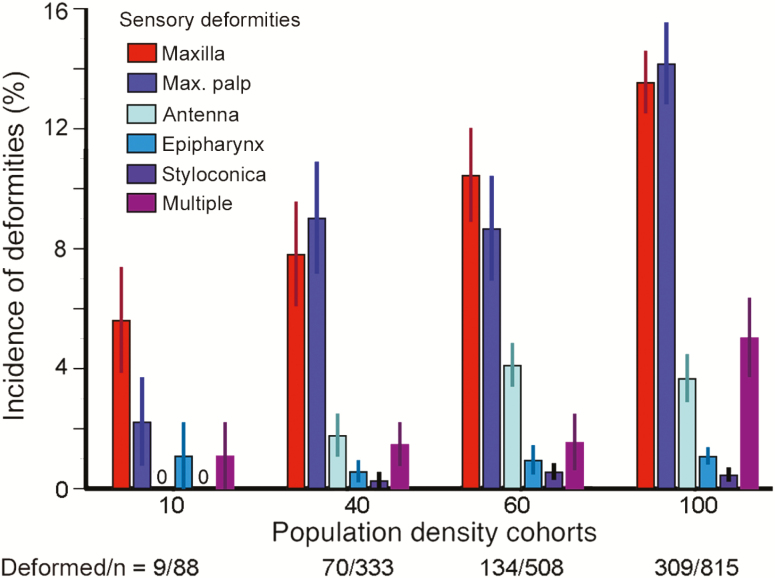
Distribution of types of deformities within each population density cohort. The height of each bar shows the incidence (% ± SE) of the designated type of deformity in that population density cohort averaged over nine trials. Deformed/*n* = total number of deformed larvae divided by the total number of surviving larvae in each density cohort summed over nine trials. Sensory defects (compare with normal in Fig. 1A): Maxilla (asterisk): deformed in Fig. 1D; Max. palp (P in Fig. 1A inset): defect is deformed P but normal styloconica (not illustrated); Antenna (diamond): missing in Fig. 1C; Epipharynx (arrow): missing left lobe in Fig. 1B; Styloconica (S in Fig. 1A inset): defect is missing or deformed S’s (not illustrated); Multiple: more than one of these defects.

**Fig. 3.**
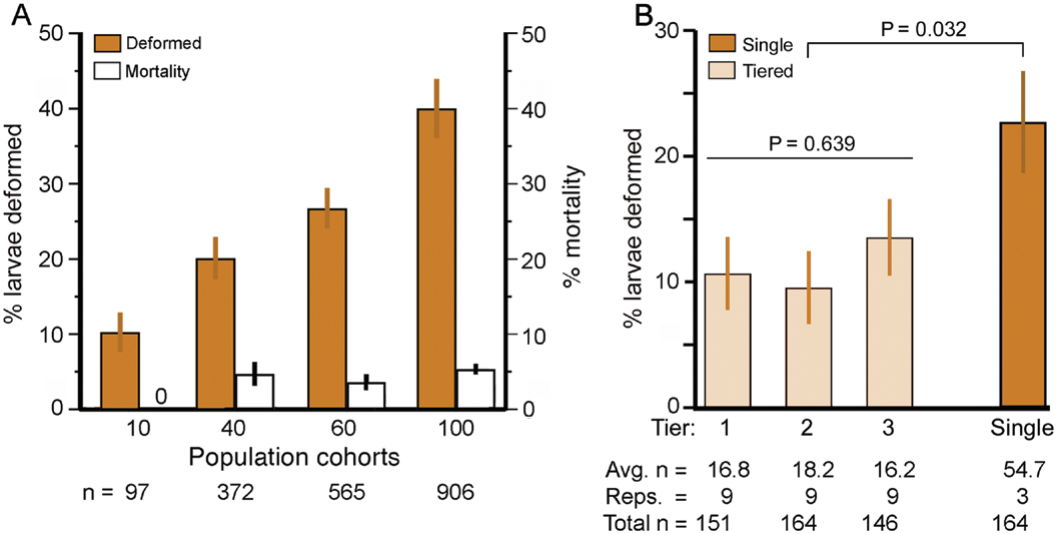
Incidence of chemosensory deformities varies directly with population density in the rearing cultures. (A) Experiment 1: Cultures reared on single feeding platforms at four different population densities. Left ordinate: incidence (% ± SE larvae deformed) of chemosensory deformities in each population density cohort (solid bars). Right ordinate: incidence of mortality (open bars). *n* = the combined number of survivors in the designated cohort summed over 10 trials. Statistical parameters are in Table 1. (B) Experiment 2: Tier platform cultures vs. single platform cultures. Incidence of chemosensory deformities in populations reared on three levels of feeding platforms stacked in a tier (pale bars) compared with populations of comparable size reared on single platforms (dark bar). Statistical parameters are in Table 2.

**Fig. 4.**
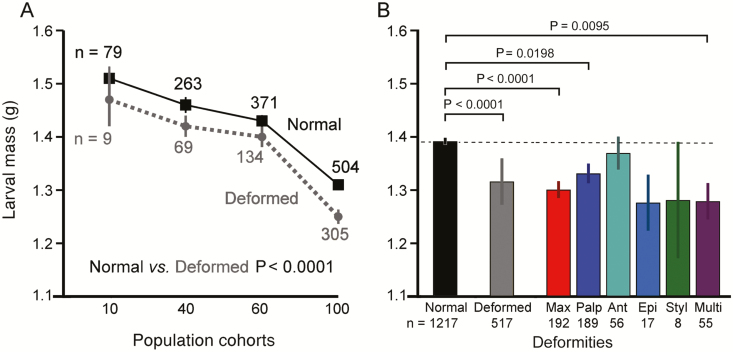
Larval mass varies with population density and chemosensory deformity. (A) Average mass (g ± SE) of larvae in each cohort. *n* = the number of normal (square symbols) or deformed (round symbols) larvae in the designated cohort summed over nine trials. The SE of some cohorts is smaller than the symbols. Statistical parameters are in Table 3. (B) Average mass (g ± SE) of normal and deformed larvae, and for each type of deformity: Max = maxilla, Palp = maxillary palp, Ant = antenna, Epi = epipharynx, Styl = maxillary styloconica, Multi = multiple chemosensory deformities. *n* = total number of larvae in the indicated category summed over nine trials. Statistical parameters are in Table 4.

**Fig. 5.**
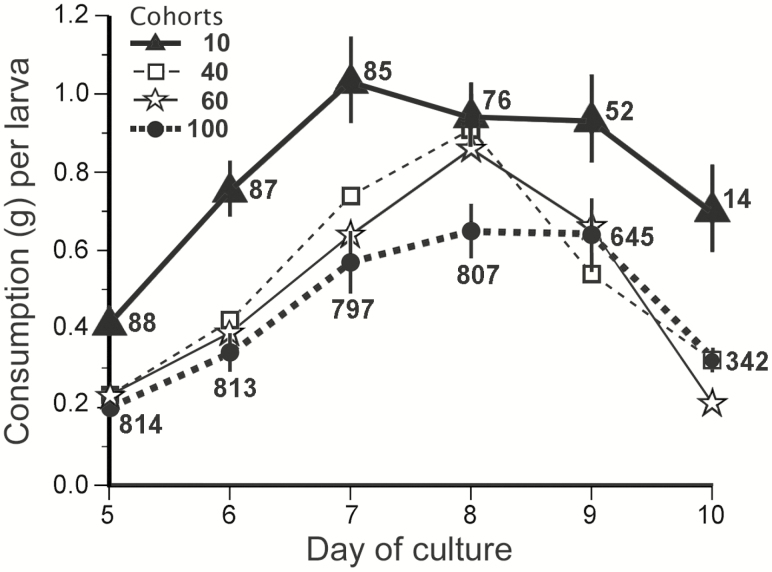
Food consumption varies inversely with population density. Symbols represent the daily consumption (g ± SE) of artificial diet per larva averaged over nine trials. Data are shown for days 5–10 of the cultures when populations were third- and fourth-instar larvae. Numbers associated with symbols of cohort densities 10 and 100 are populations on the indicated day summed over nine trials. Populations and SE bars for cohorts 40 and 60 are omitted for clarity; SE bars are similar to those of cohorts 10 and 100. Statistical parameters are in Table 5.

**Fig. 6.**
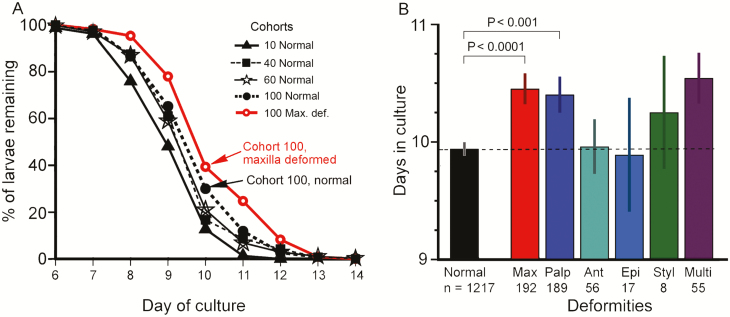
Developmental durations vary with population density and type of deformity. (A) Numbers of larvae remaining in population density cohort cultures from days 6–14 after removal of larvae that have completed development through fourth instar. Data are averaged over nine trials. Statistical parameters are in Table 6 (A). (B) The time required to reach the end of fourth instar varies with the presence and type of deformity. Bars represent the average number of days ± SE in culture to reach the end of the fourth instar. *n* = total number of larvae in the indicated category summed over nine trials. Statistical parameters are in Tables 6 (B and C).

The incidence (% of all deformed larvae) of the six types of deformities within each population density cohort was compared across the four cohorts (Fig. 2) using the chi-squared test of independence. The incidence of deformities of survivors, and the incidence of mortality, were compared across the four population density cohorts for all 10 trials using linear regressions (Fig. 3A; Table 1). In the tier platform experiment (Fig. 3B; Table 2, A), a *t*-test was used to compare the incidence of deformities in cultures reared on tier platforms versus single platforms, whereas analysis of variance (ANOVA) was employed to determine whether the incidence of deformities differed among tier platforms (Fig. 3B; Table 2, B). The relationships between larval mass versus population density, and larval mass versus deformities, were analyzed using linear regressions (Fig. 4A; Table 3). The relationship between deformity status of chemoreceptors (normal or type of deformity) and larval mass was tested using ANOVA (Fig. 4B; Table 4, A) and the relationships between specific deformities and larval mass were tested using ANOVA with Tukey’s multiple contrasts (Fig. 4B; Table 4, B). Food consumption across day of culture and population density cohorts was analyzed using linear regression (Fig. 5; Table 5). To determine whether there was a relationship between duration of development versus population density and/or deformities, we compared the percentage of larvae remaining in the culture boxes on days 6–14; the Kaplan–Meier method for analysis of survival curves and the log rank test was used to determine differences (Fig. 6A; Table 6, A). ANOVA was used to test whether duration of development varied with deformity status; ANOVA with Tukey’s multiple contrasts was used to determine if duration of development varied with each specific type of deformity (Fig. 6B; Table 6, B and C).

**Table 1.** Effect of population density on incidence of deformities and mortality

*b*_0_	SE (*b*_0_)	*b*_1_	SE (*b*_1_)	SE_Residual_	*F*	df	*P*	*R* ^2^_adjusted_
(A) Effect of population density on incidence of deformities of survivors								
0.0670	0.0287	0.0036	0.0005	0.0945	51	1,38	<0.0001	0.5626
(B) Effect of population density on incidence of mortality								
0.0009	0.0118	0.0006	0.0002	0.039	7.527	1,38	0.0092	0.1434

Statistical parameters for Fig. 3A. Linear regression: (A) Model: *y* = *b*_0_ + *b*_1_*x*_1_, where *y* = incidence of deformities (% larvae deformed), *b*_0_ = intercept, *b*_1_ = slope, *x*_1_ = population density; 1,744 larvae. (B) Model: *y* = *b*_0_ + *b*_1_*x*_1_, where *y* = incidence of mortality; 93 deaths.

**Table 2.** Tier experiment: different area densities but constant volume density

(A) Comparison of incidence of deformities on tier platforms vs. single platforms									
Variable 1	Incidence	SE	*n*	Variable 2	Incidence	SE	*n*	Test statistic	*P*
Incidence, tier platforms	11.28	2.31	461	Incidence, single platforms	22.73	4.01	164	*t* = 2.491	0.032
(B) Comparison of incidence of deformities across tier platforms									
Variable 1		No. of tier platforms		Category	No. of categories	*n*	Test statistic	df	*P*
Incidence of deformities		27		Tier	3	461	*F* = 0.457	2,24	0.639

(A) Statistical parameters for Fig. 3B. The *t*-test was used to compare incidence of deformities in larvae reared on tier platforms vs. single platforms. (B) ANOVA compared the incidence of deformities across cultures reared on the tier platforms. *n* = number of larvae.

**Table 3.** Effect of population density and chemosensory deformities on larval mass

*b*_0_	SE (*b*_0_)	*P*_0_	*b*_1_	SE (*b*_1_)	*P*_1_	*b*_2_	SE (*b*_2_)	*P*_2_	SE_Residual_	*F*	df	*P*	*R* ^2^_adjusted_
(A) Effect of population density on mass of (1) normal and (2) deformed larvae													
(1) 1.560	0.0163	<0.0001	−0.0024	0.0002	<0.0001				0.2200	125.3	1,1215	<0.0001	0.0927
(2) 1.560	0.0330	<0.0001	−0.0030	0.0004	<0.0001				0.2266	10.95	6,510	<0.0001	0.1037
(B) Combined effect of both population density and deformities on larval mass													
1.523	0.0182	<0.0001	−0.0026	0.0002	<0.0001	−0.0460	0.0118	<0.0001	0.2221	112	2,1731	<0.0001	0.1135

Statistical parameters for Fig. 4A. Linear regressions: (A) Model: *y* = *b*_0_ + *b*_1_*x*_1_, where *y* = mass (g), *b*_0_ = intercept, *b*_1_ = slope, *x*_1_ = population density. (B) Model: *y* = *b*_0_ + *b*_1_*x*_1_ + *b*_2_*x*_2_, where *b*_2_ = slope, *x*_2_ = indicator of presence of a deformity (0 = normal, 1 = deformed). *n* = 1,217 normal and 517 deformed larvae in nine trials.

**Table 4.** Effects of specific deformities on larval mass

(A) Variation in larval mass across categories of deformities						
Variable 1	*n*	Category	No. of categories	Test statistic	df	*P*
Mass	1,734	Deformity status	7	*F* = 7.298	6,1726	<0.0001
(B) Effects of specific types of deformities on larval mass						
Variable 1	*n*	Variable 2	*n*	Difference between means (g)		*P*
Mass, normal	1,217	Mass, maxilla deformity	192	−0.0898		<0.0001
Mass, normal	1,217	Mass, palp deformity	189	−0.0594		0.0198
Mass, normal	1,217	Mass, multiple deformities	55	−0.1117		0.0095

Statistical parameters for Fig. 4B. (A) ANOVA was used to compare larval mass (g) across all categories of deformity status, including normal and six types of specific deformities. (B) ANOVA with Tukey’s multiple comparisons of means was used to compare the mean mass of normal larvae vs. that of larvae having the indicated deformity. *n* = number of animals weighed in nine trials.

**Table 5.** Effect of both population density and day of culture on food consumption

*b*_0_	SE (*b*_0_)	*P*_0_	*b*_1_	SE (*b*_1_)	*P*_1_	*b*_2_	SE (*b*_2_)	*P*_2_	SE_Residual_	*F*	df	*P*	*R* ^2^_adjusted_
0.2646	0.1192	0.0274	−0.0047	0.0009	<0.0001	0.0773	0.0133	<0.0001	0.4692	25	2,248	<0.0001	0.1611

Statistical parameters for Fig. 5. Linear regression: *y* = *b*_0_ + *b*_1_*x*_1_ + *b*_2_*x*_2_, where *y* = consumption (grams of food per larva on indicated day), *b*_0_ = intercept, *b*_1,2_ = slopes, *x*_1_ = population density, *x*_2_ = day of culture. *n* = 1,743 larvae in nine trials.

**Table 6.** Effects of population density and deformities on duration of development

(A) Effects of population density on the duration of development (normal, deformed)						
Variable 1	No. of densities	Variable 2	*n*	Test statistic	df	*P*
Population density	4	Duration, normal	1,217	Log rank = 20.9	3	0.0001
Population density	4	Duration, deformed	517	Log rank = 23.7	3	<0.0001
(B) Variation in duration of development across categories of deformity status						
Variable 1	*n*	Category	No. of categories	Test statistic	df	*P*
Duration	1,734	Deformity status	7	*F* = 6.41	6,1727	<0.0001
(C) Effects of two types of deformities on duration of development						
Variable 1	*n*	Variable 2	*n*	Test statistic		*P*
Duration, normal	1,217	Duration, maxilla deformity	192	*t* = 4.759		<0.0001
Duration, normal	1,217	Duration, palp deformity	189	*t* = 3.668		<0.001

(A) Statistical parameters for Fig. 6A. The Kaplan–Meier method for analyzing survival curves was employed to compare duration of development (days) across population density for both normal and deformed larvae, and the log rank test was used to determine differences. (B) Statistical parameters for Fig. 6B. ANOVA was used to compare durations of development across all categories of deformity status, including normal and the six types of specific deformities. (C) ANOVA with Tukey’s multiple comparisons of means distinguishes between duration of development (days) for deformed larvae vs. normal larvae. Only maxilla and palp were significantly different from normals. *n* = number of larvae in nine trials.

## Results

### Chemosensory Organ Deformities: Types and Incidence

A larva with a normal set of chemosensilla and larvae with a missing or deformed epipharynx, antenna, maxilla, maxillary palp, and styloconica were selected for SEMs (Fig. 1). An experiment to determine the incidence of these deformities consisted of 10 replicate trials of rearing larvae in four population densities (10, 40, 60, and 100 per box). In the first trial, we recorded that a total of 75 of the 196 surviving larvae across all four boxes had missing or malformed chemoreceptor organs. In the subsequent nine trials (1,744 surviving larvae, 522 of which were deformed), we recorded not only the presence of the deformity, but the type of deformity as well (Fig. 2). Some larvae (~10%) had more than one type (‘Multiple’ in Figs. 2, 4, and 6). We did not find a significant association between types of deformities and population density (Fig. 2, *P* = 0.3747, chi-squared test of independence).

### Effect of Population Density on Incidence of Chemosensory Deformities and Mortality

The incidence of deformities (% of larvae deformed) increased linearly with population density (Fig. 3A; Table 1, A), ranging from 10% in the least dense cohorts of 10 larvae/box, to 39.9% in the highest density cohorts of 100 larvae/box. On average, the incidence of deformed animals increased by 3% for every 10-animal increase in cohort size. Mortality was zero in the lowest density cohort but increased to ~5% in the three higher-density cohorts (Fig. 3A; Table 1, B).

The question arises whether volatile chemicals (e.g., hypothetical stress pheromones, products of metabolism, etc.) emitted by crowded larvae may have affected the development of other larvae in their culture box. To test this, we compared the incidence of deformities of larvae reared in a high population density on a single platform versus the same number of larvae at low density in a tier of three platforms. Results showed that the incidence of deformities was significantly lower on the low-density tier platforms than on the high-density single platforms (Fig. 3B; Table 2, A). (There was no significant difference among tier platforms themselves, Table 2, B.) Thus, area-associated factors, such as physical contact between larvae, likely played more of a role in causing deformities than volume-associated factors, such as volatile chemicals.

### Effect of Population Density and Chemosensory Deformities on Larval Mass

Larval mass decreased as population density increased; this was true for both normal and deformed larvae, as well as for the combined effects of deformities and population density (Fig. 4A; Table 3). Moreover, larval mass varied with chemoreceptor deformity status (i.e., normal and types of deformities) (Table 4, A), and larvae with specific deformities of maxilla, palp, or multiple all had significantly lower mass than normal larvae (Fig. 4B; Table 4, B).

### Effect of Population Density on Larval Food Consumption

The average daily food consumption per larva changed significantly during days 5–10 of the culture (Fig. 5). Two effects are clearly visible. First, consumption increased early in this period as larvae molted into the fourth instar and began feeding; consumption peaked by days 7–8 and then decreased as larvae stopped feeding at the end of the fourth instar. These are normal feeding dynamics of larval cultures, and thus food consumption regressed onto day of culture is statistically significant as expected (Fig. 5; Table 5, *x*_2_ positive slope). The other effect, one that is more relevant to this study, is that food consumption decreased as population density increased (Fig. 5; Table 5, *x*_1_ negative slope).

### Effect of Population Density and Deformities on Duration of Development

The number of larvae in each cohort remained nearly constant until day 7 when the most rapidly developing larvae reached the end of their fourth instar and were removed as premolts (Fig. 6A). Population curves of higher-density cohorts are right-shifted towards later times, as they took longer to develop; this was true for both normal and deformed larvae (Fig. 6A; Table 6, A). ANOVA found significant variation in duration of development across deformity status categories (Fig. 6B; Table 6, B), and further analysis showed that larvae with deformities of the maxilla or maxillary palp had longer development times than normal larvae (Fig. 6B; Table 6, C).

## Discussion

In our experience with *M. sexta* larvae reared in isolation, chemosensory deformities were rarely seen. In larvae from group cultures, however, such deformities occurred frequently, and experiments showed that the incidence of missing or deformed chemosensilla was directly proportional to population density. Our data also showed that three life history traits (larval mass, food consumption, and development rates) decreased with increases in population density for both normal larvae and those with chemosensory deformities. These effects are similar to those of certain other lepidopterans, such as the speckled wood butterfly *Parage aegeria* (Gibbs et al. 2004) and the silkworm *Philo samia* (Dutta et al. 2013). Interestingly, the opposite effects were seen in larvae of the Glanville fritillary butterfly *Melitaea cinxia* which grew faster and larger when reared in high-density conditions (Rosa et al. 2017), as did larvae of the gypsy moth *Lymantria dispar* (Pavlushin et al. 2019) and the moth rice leaf roller *Cnaphalocrocis medinalis* (Yang et al. 2015). Thus, crowding effects on life history traits appear to be common phenomena, but we are unaware of any density-related occurrences of missing or deformed chemosensilla in other species.

The results of our experiments with *M. sexta* raise questions about the mechanisms responsible for these findings. First, what aspects of larval density might have led to these results? One possibility is that stressors resulting from crowding caused changes of internal chemical factors that modified normal developmental processes. In the gypsy moth *Lymantria dispar*, for example, larval crowding results in a decreased concentration of the hormone dopamine in the hemolymph (Pavlushin et al. 2019). In *Drosophila*, crowding induces heat shock proteins (reviewed by King and MacRae 2015) and affects the dopamine/ecdysteroid receptor that modulates intracellular signaling pathways in response to various stressors (reviewed by Petruccelli et al. 2020). In locusts, higher population density causes solitary-to-gregarious phase transformation, and many internal regulatory chemical agents and signal molecules are known to play a role (reviewed by Ayali 2019).

Secondly, could external chemical factors, either volatile or non-volatile, have played a role in chemosensilla alterations? There are examples of this in locusts: volatiles from frass and body odors affect the transition from solitary-to-gregarious phase (Heifetz et al. 1996, Wei et al. 2017, Ayali 2019). However, in *M. sexta*, our results from the tiered platform experiment (Fig. 3B) showed no indications of airborne volatiles causing chemosensilla deformities. More likely candidates would be non-volatile chemicals that can be transmitted by contact with other larvae. Indirect support of this alternative is found in locusts where cuticular lipids have been shown to play a role in chemical regulation of phase change (Heifetz et al. 1996, 1998).

Thirdly, could physical factors have been responsible for the effects of larval crowding? Larva-to-larva contact resulting in injury might have occurred in crowded conditions, so some chemosensilla may have been damaged during feeding in the third and fourth instars. Our data were obtained after the molt into the fifth-instar larvae, so if damage had occurred earlier, the sensory primordial tissues should have formed normal new chemosensilla during the quiescent premolt period. However, deformations were very much in evidence (Fig. 3A), so the appropriate primordial tissues must have been missing, damaged, or not activated by growth or molting hormones.

Another physical factor could be contact disturbance during feeding. This may have been responsible for the reduced consumption seen in the higher-density cohorts (Fig. 5). In general, when rates of consumption drop below the rates of protein absorption and utilization, the result is a reduction in nutrition (Woods and Kingsolver 1999). Low nutrition is known to affect insect development in many ways. For one example, three different studies on dipterans showed that larval life history traits were significantly lower for larvae reared in high densities. However, these effects were only seen when diet was limiting. When diet was not limiting, life history traits were normal (Gilles et al. 2011, Alto et al. 2012, Klepsatel et al. 2018). Thus, low nutrition, not crowding, appears to have caused these changes in life history traits.

For low nutrition to affect growth rates, larvae must be able to detect nutrient concentrations. In *Drosophila*, there are two sensing systems, one for amino acids (Colombani et al. 2003) and another for carbohydrates and lipids (Ikeya et al. 2002). The latter system is comprised of neurosecretory cells in the brain that secrete insulin-like peptides which control the growth of peripheral tissues. The two systems are hormonally linked, so any or all of these three categories of nutrients can control growth of both peripheral and primordial imaginal tissues (Koyama and Mirth 2016; reviewed by Koyama and Mirth 2018). *Manduca sexta* likely has a similar system; in low nutrition situations, it would reduce body growth and other life history traits and slow the growth of primordial tissues, presumably including those for chemosensilla which may not develop if not stimulated enough. This coordinated growth control might also explain why the incidence of chemosensilla deformations is strongly correlated with lower mass and rate of development (Figs. 4 and 6).

In addition to the above discussion of possible mechanisms, we are also mindful of feeding behavior and whether it could be affected by the loss of only one or a few chemosensilla. Reports in the literature (de Boer 1991, Flowers and Yamamoto 1992) show that the loss of half of the set of oral chemosensilla via unilateral surgical ablations resulted in some loss of food plant discrimination. Presumably, a similar behavioral deficit would be observed in larvae with the chemosensilla loss or deformity found in our experiments. The question then arises whether this could ever benefit the insect. Consider situations where oligophagous larvae in crowded rearing conditions must emigrate from a host plant that has been mostly consumed; could larvae possessing fewer functional chemoreceptors now accept some non-host plants, thereby avoiding dehydration or starvation while searching for their normal hosts? One might speculate that this could be a mechanism that evolved to loosen the tight food-choice constraints of mono- or oligophagy in situations of high population densities.

In summary, the experimental results presented here expand our understanding of the effects of larval crowding by reporting new information on another species, *M. sexta*, and on a new set of target organs, the chemosensilla. Crowding resulted in a decrease in three life history traits similar to that seen in several other species. In addition, crowding increased the incidence of missing and deformed chemosensilla, an effect which, to our knowledge, had not been previously reported. We considered possible causes and mechanisms for these effects based on research on other species, but the definitive data for *M. sexta* await future research.
